# 2024 European Society of Cardiology guidelines for the management of chronic coronary syndromes: Statement of endorsement by the NVVC

**DOI:** 10.1007/s12471-026-02059-1

**Published:** 2026-07-10

**Authors:** Alexander Hirsch, Leo H. B. Baur, Peter Damman, Marco Guglielmo, Maarten A. H. van Leeuwen, Sanne Ruigrok, Maik J. Grundeken

**Affiliations:** 1 Department of Cardiology, Cardiovascular Institute, Thorax Center, Erasmus MC, Rotterdam, The Netherlands; 2 Department of Radiology and Nuclear Medicine, Erasmus MC, Rotterdam, The Netherlands; 3 Cardiology Centres of The Netherlands, Utrecht, The Netherlands; 4 Department of Cardiology, Radboudumc, Nijmegen, The Netherlands; 5 Department of Cardiology, University Medical Center Utrecht, Utrecht, The Netherlands; 6 Department of Cardiology, Haga Teaching Hospital, The Hague, The Netherlands; 7 Department of Radiology, University Medical Centre Utrecht, Utrecht, The Netherlands; 8 Department of Cardiology, Isala Heart Center, Zwolle, The Netherlands; 9 Dutch Heart Foundation, The Hague, The Netherlands; 10 Department of Cardiology, Amsterdam University Medical Center, University of Amsterdam, Amsterdam, The Netherlands

**Keywords:** Coronary artery disease, Guidelines, Microvascular disease, Diagnostic testing/algorithm, Myocardial revascularization, Shared decision-making

## Abstract

This article discusses the updated 2024 European Society of Cardiology guidelines on chronic coronary syndromes and situates them within the context of Dutch clinical practice. A working group was appointed by the Netherlands Society of Cardiology (Nederlandse Vereniging voor Cardiologie). It consisted of a general cardiologist, interventional cardiologists, cardiovascular imaging cardiologists, and a representative of the Dutch Heart Foundation (Nederlandse Hartstichting). This document summarizes the key recommendations and includes additional guidance tailored to clinical practice in the Netherlands, focusing on the risk-factor-weighted clinical likelihood model, the choice of non-invasive imaging modality, functional assessment of epicardial artery stenosis during invasive coronary angiography, and angina and ischemia with non-obstructive coronary arteries. It is intended for all healthcare professionals involved in the care of patients with chronic coronary syndromes and has been approved by the Netherlands Society of Cardiology and the Dutch Heart Foundation.

## Introduction

In cardiology, chest pain is among the most common reasons for outpatient clinic visits and remains a challenge for physicians. To guide the management of patients with stable chest pain in the outpatient setting, the European Society of Cardiology (ESC) has updated the guidelines for stable coronary artery disease (CAD). In 2019, the writing committee of the ESC guidelines introduced the term chronic coronary syndromes (CCS) to describe the clinical presentations of CAD in a stable setting [1]. The ESC guidelines for CCS were updated in 2024 [2]. The guidelines are endorsed by the European Association for Cardio-Thoracic Surgery.

The Netherlands Society of Cardiology (Nederlandse Vereniging voor Cardiologie; NVVC) has established a working group to assess the 2024 ESC Guidelines on CCS in relation to everyday clinical practice in the Netherlands and to identify which recommendations can be fully endorsed and which should be modified or excluded. This effort has led to an amendment to the ESC guidelines, intended for all healthcare professionals who care for patients with CCS, and it has received approval from the NVVC and the Dutch Heart Foundation.

## Methods

### Composition of the working group:

To compose this addendum, a working group was appointed in 2024 comprising a general cardiologist, interventional cardiologists, cardiovascular imaging cardiologists, and a representative of the Dutch Heart Foundation (previously known as Harteraad). Cardiologists from both academic and top clinical hospitals, as well as an independent cardiology treatment center, participated, ensuring optimal regional distribution.

### Declarations of interest:

The working group has adhered to the Royal Dutch Medical Association (Koninklijke Nederlandsche Maatschappij tot bevordering der Geneeskunst) code of conduct on preventing undue influence due to conflicts of interest. All members of this working group have disclosed any potential conflicts of interest from the past three years.

### Drafting of this addendum:

The working group assessed all recommendations from the guideline for applicability in the Netherlands. All recommendations were assessed during several meetings. In particular, recommendations with a low level of evidence (*level of evidence C*; *expert opinion*) are assessed for applicability in the Dutch clinical setting. Recommendations with a higher level of evidence can be changed only in light of new literature or changes in Dutch cardiology practice. Any modifications to the recommendations were agreed upon by consensus. An overview highlighting several comments from the working group is depicted in Tab. 1.

**Table 1:** European Society of Cardiology guideline recommendations adopted into clinical practice in the Netherlands, highlighting several comments from the working group discussed in this endorsement paper.

*ESC guideline*	*Class*	*LOE*	*Table*	*Comment from the working group*
Recommendation Chapter 3.2.1.				
It is recommended to use additional clinical data (e.g. examination of peripheral arteries, resting ECG, resting echocardiography, presence of vascular calcifications on previously performed imaging tests) to adjust the estimate yielded by the Risk Factor-weighted Clinical Likelihood model	I	C	3	– Although we agree that additional clinical data can change the clinical likelihood, the original paper [7] and the guideline are unclear about the extent to which these factors influence the clinical likelihood and, therefore, how these changes will affect subsequent diagnostic steps
In individuals with a low (> 5%–15%) pre-test likelihood of obstructive CAD, CAC score should be considered to reclassify subjects and to identify more individuals with very low (≤ 5%) CAC score-weighted clinical likelihood	IIa	B	3	– This aligns with earlier Dutch recommendations [3]. However, this working group notes that the added diagnostic value of a CAC score of 0 is lower in younger patients compared to older patients – Therefore, it is important to consider further diagnostic evaluation, particularly in younger patients with persistent symptoms without an alternative explanation, despite a CAC score of 0
Recommendation Chapter 3.2.2.				
A resting transthoracic echocardiogram is recommended: – to measure left ventricular ejection fraction, volumes and diastolic function – to identify regional wall motion abnormalities – to identify non-coronary cardiac disease; – to assess right ventricular function and estimate systolic pulmonary artery pressure; – to refine risk stratification and guide treatment	I	B	4	– This working group emphasizes the additional value of ventricular function information for risk assessment in patients with CAD – However, routine echocardiography for all patients presenting with chest pain in the outpatient setting may have limited value if the physical exam and ECG are normal. Additionally, it depends on whether a non-invasive imaging test will be performed and which one, e.g. cardiovascular magnetic resonance or positron emission tomography – When clinically indicated, imaging techniques should be performed to exclude alternative causes of chest pain, but not to estimate the pre-test probability of obstructive CAD [3]
Recommendation Chapter 3.3.1.				
In individuals with suspected CCS and low or moderate (> 5%–50%) pre-test likelihood of obstructive CAD, CCTA is recommended to diagnose obstructive CAD and to estimate the risk of MACE	I	A	8	– One disadvantage of CCTA is its relatively moderate positive predictive value, particularly in patients with heavily calcified plaques. Therefore, CCTA is less suitable in patients with a high pre-test likelihood of obstructive CAD – However, with ongoing technological advancements—including photon-counting CT—this is expected to further improve – The choice between CCTA and functional tests depends on practical factors like cost, availability, and local technology and expertise, and may therefore also be considered in patients with an estimated pre-test likelihood of 50–85%
Recommendation Chapter 3.3.3.				
When ICA is indicated, it is recommended to have coronary pressure assessment available and to use it to evaluate the functional severity of intermediate non-left main stem stenoses prior to revascularization	I	A	11	– This is different from the Dutch situation, in which a considerable proportion of ICA are performed in non-PCI centers. The question if all ICAs should be performed in dedicated intervention centers is beyond the scope of this endorsement statement – However, this working group wishes to emphasize that the RF-CL should be used in combination with appropriate non-invasive imaging techniques to ensure optimal patient selection for referral for ICA – When ICA is performed in a PCI center, coronary pressure wires should be readily available to assess functional severity during the same ICA
Recommendation Chapter 3.3.3.				
During ICA, selective assessment of functional severity of intermediate diameter stenoses is recommended to guide the decision to revascularize, using the following techniques: – QFR (significant ≤ 0.8)	I	B	12	– FFR or iFR should be the preferred method over QFR for the assessment of intermediate stenoses when both modalities are available and can be performed [25]

*CAC score* coronary artery calcium score, *CAD* coronary artery disease, *CCS* chronic coronary syndrome, *CCTA* coronary computed tomography angiography, *ECG* electrocardiogram, *ESC* European Society of Cardiology, *FFR* fractional flow reserve, *ICA* invasive coronary angiography, *iFR* instantaneous wave-free ratio, *MACE* major adverse cardiac event, *PCI* percutaneous coronary intervention, *QFR* quantitative flow ratio

### Comment and authorization phase:

A first draft of this addendum was submitted to the members of the NVVC and the Dutch Heart Foundation. Collected comments were discussed within the working group. The initial draft was revised and resubmitted to the NVVC and the Dutch Heart Foundation for final approval.

## Summary and translation into clinical practice in the Netherlands

CCS is conceptualized in the 2024 ESC guideline as a broad, dynamic set of clinical presentations that arise from structural and/or functional abnormalities of the coronary circulation and microvasculature, and that can produce a transient mismatch between myocardial oxygen supply and demand. This mismatch commonly manifests as exertional angina, chest discomfort, or exertional dyspnea, but many presentations are non-classical and may be asymptomatic. Importantly, the underlying CAD is often progressive and may destabilize at any time with transition to an acute coronary syndrome. The guideline, therefore, frames ‘disease’ as the underlying coronary pathology and ‘syndrome’ as the clinical manifestation, and it explicitly expands older, narrower concepts of stable angina to include diffuse atherosclerosis without focal luminal narrowing, myocardial bridging, dynamic epicardial vasospasm, and coronary microvascular dysfunction as frequent and clinically important mechanisms of ischemia. This broader pathophysiological view underpins the diagnostic and therapeutic recommendations throughout the document [2].

### The stepwise approach to the initial management of individuals with suspected chronic coronary syndrome

#### Step 1: General clinical examination

Initial evaluation of a person with symptoms suggestive of myocardial ischemia begins with a careful, structured clinical assessment including a 12-lead resting electrocardiogram (ECG) and basic blood tests. The guideline places renewed emphasis on detailed history taking that documents the onset, duration, quality, location, triggers, relieving factors, and temporal pattern of symptoms. It highlights a wider range of anginal equivalents, for example, chest pain triggered by emotional stress, exertional dizziness or fatigue, and pain in the jaw, neck, arms, or upper back, that should prompt consideration of myocardial ischemia (*Class IIa, level of evidence B*). It is important to thoroughly evaluate chest pain, including an objective exclusion of myocardial ischemia caused by obstructive CAD, microvascular disease, and/or coronary vasospasm, before classifying it as non-cardiac. These recommendations align with Dutch guidelines and recommendations [3–5].

#### Step 2: Further evaluation

**Fig. 1:**
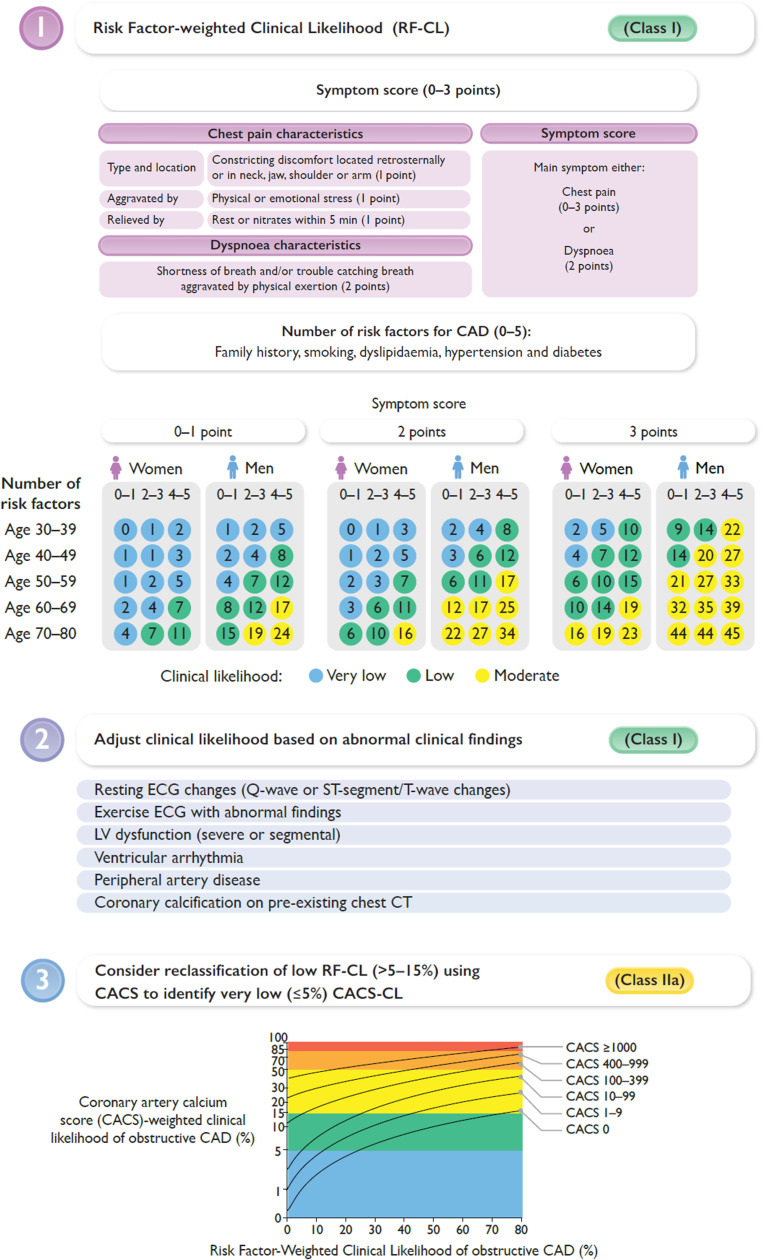
Estimation of the clinical likelihood of obstructive coronary artery disease based on the Risk Factor-weighted Clinical Likelihood model from Winther et al. [2, 6]. A combination of Figures 4 and 5 as published in the 2024 ESC guideline of chronic coronary syndromes (reproduced with the permission of Oxford University Press) [2]. *CACS* coronary artery calcium score, *CACS-CL* coronary artery calcium score + RF-CL model, *CAD* coronary artery disease, *CT* computed tomography, *ECG* electrocardiogram, *LV* left ventricular, *RF-CL* risk factor-weighted clinical likelihood

A central methodological innovation of the 2024 guideline is the adoption of the Risk Factor-weighted Clinical Likelihood (RF-CL) model from Winther et al. [6] to estimate the pre-test probability of obstructive epicardial CAD, rather than the Diamond-Forrester approach used in the 2019 guidelines [1]. Unlike the older symptom-only classifications, the RF-CL integrates age, sex, a standardized symptom score, and the number of traditional coronary risk factors (smoking, hypertension, diabetes, dyslipidemia, and family history). The RF-CL model, which incorporates risk factors, improves the prediction of obstructive CAD, downclassifies more individuals to ‘very low’ and ‘low’ likelihood of CAD, and maintains its high calibration (Fig. 1). The RF-CL thus produces a graded clinical likelihood that guides further testing. These refinements reflect the declining prevalence of obstructive CAD in contemporary cohorts and aim to reduce over-investigation while preserving sensitivity for significant disease.

Interestingly, when the RF-CL model is used, patients will never be classified with a clinical likelihood greater than 45%, which is considered a ‘moderate’ likelihood (> 15%–50%).

Furthermore, the guideline also recommends using readily available clinical data (resting or exercise ECG or echocardiographic abnormalities, ventricular arrhythmias, peripheral artery disease, or coronary calcification on pre-existing chest Computed Tomography (CT)) to adjust RF-CL estimates (*Class I recommendation, level of evidence C*) (point 2 in Fig. 1). However, the guidelines are unclear regarding how the presence of these factors influences the RF-CL estimates. The study they cite only considers the risk factors already accounted for in the RF-CL model [7]. It is therefore unclear when a patient will be considered high-risk (50%–85%) or very high-risk (> 85%). This is important because the pre-test likelihood determines the appropriate first-line test for symptomatic individuals, as described in step 3 (confirming the diagnosis).

In individuals with a low likelihood (> 5%–15%), it should be considered to use the coronary artery calcium (CAC) score to reclassify subjects to very low risk (< 5%) when the CAC score is 0 (*Class IIa recommendation, level of evidence B*) (point 3 in Fig. 1). This aligns with earlier Dutch recommendations [3] and is supported by this working group. However, this working group notes that the diagnostic value of a CAC score of 0 is lower in younger patients compared to older patients. In a study by Mortensen et al., obstructive CAD was rare in patients with a CAC score of 0 (6%), especially in younger patients. However, when CAD was present, a higher percentage of younger patients had a CAC score of 0 (58% of those younger than 40 years) compared with older patients with obstructive CAD (9% among those aged 60 to 69 years) [8]. This finding was observed in both men and women separately. However, throughout the age spectrum, a substantially higher proportion of women with obstructive CAD had a CAC score of 0 as compared with men. Therefore, it is important to consider further diagnostic evaluation, particularly in younger patients with persistent symptoms without an alternative explanation, despite a CAC score of 0.

The ESC guideline committee recommends performing a transthoracic echocardiography in all patients with chest pain. This working group emphasizes the additional value of ventricular function information for risk assessment in patients with CAD. However, routine echocardiography for all patients presenting with chest pain in the outpatient setting may have limited value if the physical exam and ECG are normal. Additionally, it depends on whether a non-invasive imaging test will be performed and which one, e.g., cardiovascular magnetic resonance (CMR) or positron emission tomography (PET) myocardial perfusion imaging (see step 3). However, when clinically indicated, imaging techniques should be performed to exclude alternative causes of chest pain, but not to estimate the pre-test probability of obstructive CAD [3].

#### Step 3: Confirming the diagnosis

When non-invasive testing is indicated, the guideline recommends a pragmatic, availability- and expertise-sensitive approach. Coronary computed tomography angiography (CCTA) is strongly advocated as a first-line anatomical test in many patients with low to moderate clinical likelihood (> 5–50%), because of its high negative predictive value and its unique ability to visualize both obstructive and non-obstructive plaque, to provide prognostically informative plaque phenotyping, and to tailor patient management [2, 9]. One disadvantage of CCTA is its relatively moderate positive predictive value, particularly in patients with heavily calcified plaques. However, with ongoing technological advancements—including photon-counting CT—this is expected to further improve [10, 11]. Furthermore, CCTA, when combined with computational tools such as Fractional Flow Reserve (FFR)-CT, may discriminate hemodynamically significant lesions, thereby improving patient selection for ICA. To investigate the impact of adding FFR-CT analysis to the diagnostic pathway in patients with a coronary stenosis on CCTA on the rate of unnecessary invasive coronary angiography (ICA), cost-effectiveness, and quality of life in the Netherlands, the FUSION (‘Addition of FFRct in the diagnostic pathway of patients with stable chest pain to reduce unnecessary invasive coronary angiography’, NCT05174247) trial was designed [12]. The results are expected in 2026. In light of ongoing technological advances, the working group underscores that CCTA can be considered in patients with increasingly higher a priori risk, depending on local expertise and equipment availability.

Functional stress imaging options retain an important role, particularly when ischemia quantification is required or when the primary clinical question concerns myocardial viability or perfusion. In the ESC guideline, functional stress imaging with either stress echocardiography, single-photon emission computed tomography, PET, or CMR perfusion, is considered appropriate in patients with moderate to high clinical likelihood (> 15–85%). The guideline does not recommend one non-invasive functional test over another, and the choice of modality should be based on local expertise and availability. This working group endorses this statement, noting that PET and CMR perfusion generally demonstrate higher diagnostic accuracy than single-photon emission computed tomography for detecting flow-limiting stenosis on ICA, and further emphasizes that stress echocardiography should be performed only by experienced operators with high annual volumes [13–15]. Thus, the selection of the initial test is individualized based on RF-CL, local resources, comorbidities, and patient preference. Active patient involvement in shared decision-making is essential when selecting among different diagnostic tests and when considering whether to forgo additional diagnostic evaluation.

The ESC guideline recommends functional imaging for myocardial ischemia if CCTA has shown CAD of uncertain functional significance (*Class I recommendation, level of evidence B*). In the 2017 ‘Improvement Report on Chest Pain’ (Verbetersignalement pijn op de borst) by the National Health Care Institute of the Netherlands (Zorginstituut Nederland), the use of a single diagnostic test is advocated rather than the sequential use of multiple non-invasive investigations [16]. Although the addition of a functional non-invasive test to CCTA (hybrid cardiac imaging) demonstrates improved diagnostic specificity for the detection of obstructive CAD compared with stand-alone coronary CCTA, the improvement in overall diagnostic performance is relatively limited [17]. Rasmussen et al. reported that, in patients with suspected obstructive stenosis at CCTA, CMR and PET perfusion had similar, moderate sensitivities (59% and 64%, respectively) but high specificities (84% and 89%, respectively) compared with ICA with FFR [18]. Therefore, patients with suspected obstructive stenosis at CCTA represent a diagnostic challenge, with frequent mismatch between advanced myocardial perfusion tests and invasive measurements [18]. As a working group, we agree with the ESC guideline that functional testing in patients with suspected obstructive stenosis on CCTA provides important prognostic information [19–21]; however, it remains questionable whether this approach adequately identifies the correct patients for ICA and is the most cost-effective. In this light, it is important to refer to the CLEAR-CAD trial (‘Clinical Outcomes and Cost-effectiveness of a Diagnostic and Treatment Strategy of Upfront CTCA plus Selective Non-Invasive Functional Imaging Compared with Standard Care in Patients with Chest Pain and Suspected CAD’, NCT05344612) that is currently running in the Netherlands. This study plans to include 6444 patients randomized to an upfront CCTA strategy and subsequent non-invasive functional testing if CCTA reveals significant obstructive CAD, compared with the standard of care [22]. Inclusion is expected to be finished by the end of 2026, and the first trial results are expected in 2028. Pending these results, the working group discourages the sequential use of multiple non-invasive tests before proceeding to ICA.

ICA is recommended when the clinical likelihood of obstructive CAD is very high (> 85%), when non-invasive tests indicate high-risk anatomy or extensive ischemia, or when symptoms are refractory despite optimal medical therapy, and revascularization is being considered.

The ESC guideline emphasizes that ICA should be paired with physiological lesion assessment (FFR, instantaneous Wave-Free Ratio (iFR)) or angiography-derived surrogates (Quantitative Flow Ratio (QFR)) for intermediate stenoses to identify functionally significant stenoses and guide revascularization decisions. This is different from the Dutch situation, in which a considerable proportion of ICAs are performed in non-percutaneous coronary intervention (PCI) centers. The question of whether all ICAs should be performed in dedicated intervention centers is beyond the scope of this endorsement statement. However, this working group wishes to emphasize that the RF-CL should be used in combination with appropriate non-invasive imaging techniques to ensure optimal patient selection for referral for ICA. Furthermore, patients should be referred to PCI centers for invasive pressure measurements when indicated. All PCI centers should be equipped with pressure wires.

The ESC guideline recommends iFR or FFR (both as *Class I, level of evidence A*) recommendation for assessing intermediate stenosis based on abundant evidence, including a comparative randomized trial [23]. The ESC guideline recommends QFR, a wireless method to assess functional severity by using a three-dimensional reconstruction of the angiography-derived coronary artery, with an equal *Class I* recommendation to assess functional significance of intermediate lesions, based on the superiority of QFR compared to angiography in the FAVOR III (‘Functional Assessment by Virtual Online Reconstruction’) China trial [24]. This working group, however, believes the recommendation was made too prematurely, as data on a direct comparison with either FFR or iFR were lacking. After publication of the guideline, the recent FAVOR III Europe randomized trial failed to demonstrate non-inferiority of QFR compared with FFR [25]. In the same trial, FFR was even shown to be superior to QFR. Therefore, QFR is not recommended when FFR or iFR is available and can be performed, although it is reasonable to consider QFR to avoid a second ICA in case the first ICA was performed in a non-PCI center or in a scenario in which FFR or iFR are not available (*Class IIb recommendation* for QFR). Recently, preliminary results of the PIONEER IV (‘Non-inferiority of Angiography-derived Physiology Guidance Versus Usual Care in an All-comers PCI Population’) trial were presented at the ESC Congress 2025 in Madrid, Spain. In this trial, QFR was non-inferior compared to a ‘standard of care’ arm in which only a minority of patients (41%) were interrogated using physiology [26]. More trials are needed to establish the exact position of QFR in the field of physiology-based interrogation of intermediate coronary lesions.

#### Step 4: Initial therapy

Initial therapy, including beta-blockers, short-acting nitrates, anti-anginal medication, and preventive medication, can be started immediately, during, or after the diagnostic process, based on anticipated risk of adverse events, symptoms, and patient preference.

### Guideline-directed therapy

Guideline-directed therapy for CCS has dual aims: improving quality of life by relieving angina and reducing the risk of cardiovascular death, myocardial infarction, and disease progression.

#### Patient education, lifestyle optimization for risk-factor control, and exercise therapy

The ESC guidelines stress the importance of patient education, lifestyle optimization for risk-factor control, and exercise therapy, and this working group endorses the associated recommendations. Also in the revised 2024 Dutch guideline from the Federation of Medical Specialists, cardiac rehabilitation is recommended for patients with stable CAD [27]. However, as of March 2026, the National Health Care Institute of the Netherlands (Zorginstituut Nederland) no longer reimburses cardiac rehabilitation for stable angina pectoris, citing a lack of evidence on effectiveness [28]. We emphasize that it is important to further investigate the possibilities for reimbursement in the Netherlands, including Combined Lifestyle Intervention, when indicated.

#### Anti-anginal/anti-ischemic medication

Symptom control is individualized and frequently requires combination anti-anginal therapy aimed at reducing myocardial oxygen demand or improving coronary blood flow. The ESC guideline recommends tailoring anti-anginal drugs to patient-specific characteristics, the underlying etiology of angina, comorbidities, other medications, treatment tolerability, and patient preference, while considering availability and costs. There are no large randomized trials that can guide us in choosing the appropriate first-choice anti-anginal medication. No head-to-head comparative studies are available between first-generation and newer-generation anti-anginal drugs. There is no evidence of improved survival with anti-anginal drugs (except for beta-blockers after acute myocardial infarction). This working group endorses the recommendations and emphasizes the importance of shared decision-making. There’s a great need for patients to be included in the process of tailoring anti-anginal drugs. When patients feel they can discuss their treatment with the physician, they are more likely to accept and adhere to the chosen option. This also strengthens trust in the professional, self-efficacy, and confidence to ask questions and be critical. It is important for the healthcare professional to improve medication and treatment adherence. The working group notes that some anti-anginal medications mentioned in the ESC guidelines are (currently) not available in the Netherlands, like ranolazine and trimetazidine; however, there are sufficient alternatives.

#### Medical therapy for event prevention

This working group endorses the recommendations on antiplatelet therapy, lipid-lowering therapy, and other preventive medications as stated in the guideline. Regarding the recommended lipid-lowering targets, we refer to the 2024 Dutch Multidisciplinary Guidelines for Cardiovascular Risk Management [29]. We note that in CCS patients with a prior myocardial infarction or PCI, clopidogrel 75 mg daily is recommended as a safe and effective alternative to aspirin monotherapy. The benefit of clopidogrel 75 mg daily, compared to aspirin, is of debatable clinical relevance, in view of the high number needed to treat to prevent a myocardial infarction and the absence of any effect on all-cause and vascular mortality [30].

#### Revascularization for chronic coronary syndromes

When revascularization is being considered, strategies should be based on shared decision-making, with patients being adequately informed about the potential benefits and risks, therapeutic implications, and alternative treatment options. When both PCI and coronary artery bypass grafting are reasonable options, multidisciplinary consultation involving an interventional cardiologist and a cardiac surgeon is recommended. In patients with CCS with obstructive coronary artery disease, revascularization should primarily aim to relieve anginal symptoms in patients with refractory symptoms despite optimized guideline-directed medical treatment, although in selected subgroups, revascularization may also confer a prognostic benefit. The choice of revascularization modality (PCI versus coronary artery bypass grafting) should take into account surgical risk, the complexity and extent of CAD, comorbidities (including diabetes mellitus), left ventricular function, expected completeness of revascularization, the need for concomitant surgical procedures, and patient preference.

### Optimal assessment and treatment of specific groups

#### Coronary artery disease and heart failure

This working group endorses the view that optimal medical therapy for heart failure is the cornerstone of treatment. In about 50% of patients with reduced left ventricular systolic function, the etiology is ischemic. The ESC guideline recommends ICA as the preferred modality to diagnose an underlying ischemic etiology. However, this working group would like to stress that, given the likelihood of an ischemic origin, other modalities may be used first to differentiate among etiologies, such as CCTA in cases with low likelihood of obstructive CAD or CMR when a specific cardiomyopathy is suspected. Furthermore, it is important to emphasize that the REVIVED-BCIS2 (‘Percutaneous Revascularization for Ischemic Left Ventricular Dysfunction’) trial did not demonstrate a prognostic benefit of PCI revascularization in patients with severe ischemic left ventricular systolic dysfunction, as compared with optimal medical therapy [31].

#### Angina/ischemia with non-obstructive coronary arteries

A particularly important and expanded area of the guideline concerns angina and ischemia with non-obstructive coronary arteries (ANOCA/INOCA). The guideline acknowledges that a large fraction of patients referred for invasive assessment have non-obstructive epicardial arteries yet exhibit demonstrable myocardial ischemia or angina. Coronary vasomotor dysfunction is the underlying cause of INOCA in up to almost 90% of patients, encompassing the endotypes of coronary microvascular dysfunction and epicardial coronary vasospasm [32]. Coronary vasomotor dysfunction and the underlying endotype are diagnosed by invasive coronary vasomotor function testing.

**Fig. 2:**
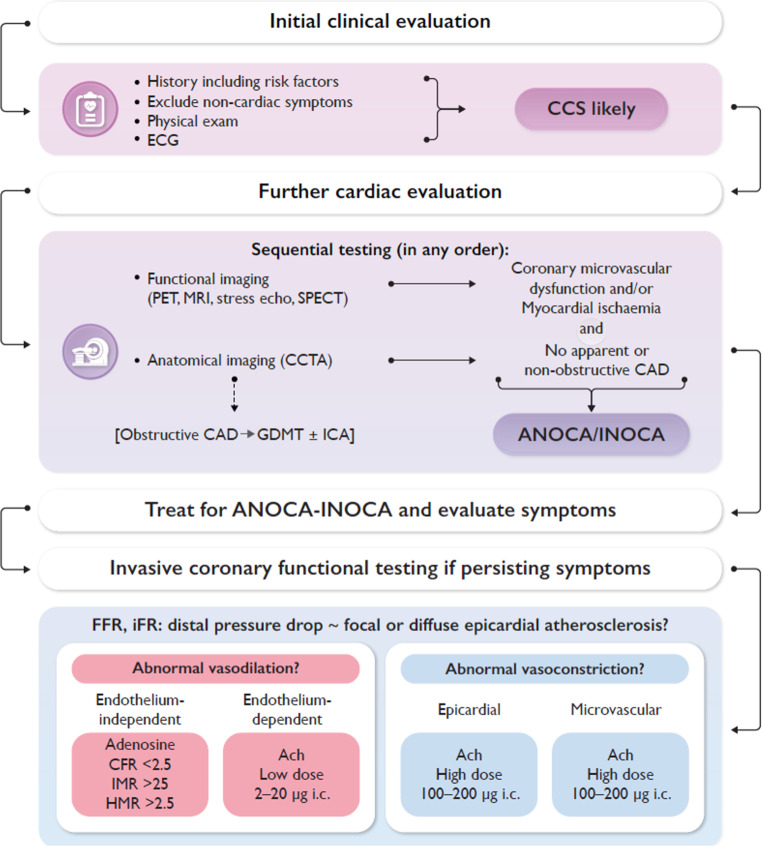
Diagnostic algorithm for patients with angina/ischemia with non-obstructive coronary arteries. Published in the 2024 ESC guideline of chronic coronary syndromes (Fig. 13, reproduced with the permission of Oxford University Press) [2]. *Ach* acetylcholine, *ANOCA* angina with non-obstructive coronary arteries, *CAD* coronary artery disease, *CCS* chronic coronary syndrome, *CCTA* coronary computed tomography angiography, *CFR* coronary flow reserve, *ECG* electrocardiogram, *echo* echocardiography; *FFR* fractional flow reserve, *GDMT* guideline-directed medical therapy, *HMR* hyperemic myocardial velocity resistance, *i.c.* intracoronary, *ICA* invasive coronary angiography, *iFR* instantaneous-wave free ratio, *IMR* index of microcirculatory resistance, *INOCA* ischemia with non-obstructive coronary arteries, *MRI* magnetic resonance imaging, *PET* positron emission tomography, *SPECT* single-photon emission computed tomography

Current ESC guidelines recommend coronary vasomotor function testing in persistently symptomatic patients despite medical treatment, with suspected ANOCA/INOCA (i.e., anginal symptoms with normal coronary arteries or proven non-obstructive lesions by non-invasive imaging or (intra)coronary physiology and poor quality of life) (Fig. 2). Two studies have shown that coronary vasomotor function testing -guided treatment improves symptoms and quality of life [33, 34]. Additionally, it has been shown that coronary vasomotor function testing is feasible and safe to perform in the Netherlands [35]. The ESC guideline recommends considering patient choices and preferences. A tool for shared decision-making can be used to assist in this process, such as the Coronary Functional Test Decision Aid (‘Keuzehulp Coronaire Functietest’) [36]. In cases where coronary vasomotor function testing is not feasible or preferred, the ESC guideline recommends that transthoracic Doppler of the left anterior descending artery or functional noninvasive imaging may be considered to assess coronary microvascular dysfunction by measuring coronary flow reserve. However, this working group would stress that the preferred non-invasive imaging technique is PET perfusion, although it is costly and not widely available. The other non-invasive imaging techniques should be performed only in centers with clear expertise and sufficient exposure. Furthermore, we note that vasospasm is not assessed with these methods. For vasospastic angina, a resting 12-lead ECG recording during angina is recommended, or ambulatory ECG monitoring should be considered to identify ST-segment deviation during angina.

The management of coronary vasomotor dysfunction includes statins and angiotensin-converting enzyme inhibitors, and (combinations of) anti-anginal medication.

### Patient-centered care and shared decision-making

Variations in the patients’ daily life, new or different symptoms, and changes from branded to generic medicines can lead to new or different treatments. Therefore, long-term treatment of CCSs requires constant evaluation and optimization. To make decisions on diagnostics and treatment in a shared manner. For every alteration of the treatment plan, personal preferences and (dis)advantages should be considered. Given the heterogeneity inherent to CCSs, which results in substantial variability in patients’ symptoms, disease burden, and acceptance of their condition, a clinical environment should be established that enables an open and candid discussion between the patient and healthcare provider about the burden of disease. Factors that can contribute to creating an appropriate setting for honest discussions include explicit agreements between the patient and the health care provider, accessible forms of communication, and planned evaluations. The working group wishes to emphasize that both patients and healthcare professionals share responsibility for achieving well-informed shared decision-making.

## Conclusion

Taken together, the 2024 ESC guideline reframes CCS as a spectrum disorder driven by both structural and functional coronary abnormalities and promotes an individualized diagnostic approach anchored in the RF-CL model and selective use of CCTA and functional imaging. Furthermore, the guideline mandates mechanistic evaluation for patients with persistent symptoms without obstructive epicardial CAD and emphasizes a dual therapeutic focus on symptom relief and rigorous secondary prevention. The guideline balances pragmatic, resource-sensitive recommendations with precise procedural guidance for invasive physiology and imaging, and it reinforces patient-centered shared decision-making across diagnostic and treatment choices.
